# Uncemented hip arthroplasty and denosumab: increased postoperative dipeptide concentrations and identification of potential new bone turnover biomarkers

**DOI:** 10.1093/jbmrpl/ziaf091

**Published:** 2025-05-19

**Authors:** Kim Kultima, Saman Hosseini Ashtiani, Ida Erngren, Payam Emami Khoonsari, Henrik Carlsson, Stephanie Herman, Eva Freyhult, Hans Mallmin, Nils P Hailer

**Affiliations:** Department of Medical Sciences, Clinical Chemistry, Uppsala University, 751 85 Uppsala, Sweden; Department of Medical Sciences, Clinical Chemistry, Uppsala University, 751 85 Uppsala, Sweden; Science for Life Laboratory, Department of Biochemistry and Biophysics, Stockholm University, 106 91 Stockholm, Sweden; Department of Medical Sciences, Clinical Chemistry, Uppsala University, 751 85 Uppsala, Sweden; Department of Medical Sciences, Clinical Chemistry, Uppsala University, 751 85 Uppsala, Sweden; Department of Medical Sciences, Clinical Chemistry, Uppsala University, 751 85 Uppsala, Sweden; Department of Medical Sciences, Clinical Chemistry, Uppsala University, 751 85 Uppsala, Sweden; Department of Cell and Molecular Biology, Uppsala University, 751 24 Uppsala, Sweden; Department of Surgical Sciences/Orthopaedics, Uppsala University, 751 85 Uppsala, Sweden; Department of Surgical Sciences/Orthopaedics, Uppsala University, 751 85 Uppsala, Sweden

**Keywords:** denosumab, bone turnover biomarkers, total hip arthroplasty, rebound bone loss, metabolomics, osteoclast activity, dipeptides, high-resolution mass spectrometry, periprosthetic bone loss

## Abstract

Denosumab is a potent antagonist of RANKL and is widely used to treat severe postmenopausal osteoporosis. Using high-resolution mass spectrometry (HRMS), we aimed to identify molecular mediators associated with the rapid reactivation of osteoclasts following discontinuation of denosumab. In a previously reported randomized controlled trial, 64 patients undergoing uncemented total hip arthroplasty were randomized to receive 2 doses of 60 mg denosumab or placebo, administered 1-3 d and 6 mo postoperatively. Serum samples were analyzed using untargeted HRMS coupled with liquid chromatography, and bone turnover markers were assessed. Data were evaluated using linear mixed-effects models and machine learning techniques. After surgery, 83 metabolite features showed significant concentration changes (*p* < .0001). Denosumab-treated patients exhibited increased levels of the dipeptides di-L-phenylalanine, phenylalanylleucine, and alpha-Asp-Phe, and decreased levels of fibrinopeptide A and related peptides 24 mo after surgery. The oxidized peptide AP(Ox)GDRGEP(Ox)GPP(Ox)GP, derived from the collagen type I alpha 1 chain (COL1A1) and referred to as COL1A1-OxP, showed a strong correlation with the bone formation marker procollagen type 1 amino-terminal propeptide (P1NP) (*p* = 4.4E^−83^). Similarly, the tripeptide DL-alpha-aspartyl-DL-valyl-DL-proline (DVP) correlated highly with the bone resorption marker carboxy-terminal telopeptide of type 1 collagen (CTX) (*p* = 1.1E^−222^). P1NP and CTX levels were suppressed at 3, 6, and 12 mo postoperatively but exceeded baseline levels by 24 mo. Global metabolic shifts were observed postoperatively, with distinct profiles between treatment groups. The observed increase in specific dipeptides may reflect mechanisms contributing to rebound bone loss following denosumab withdrawal. Fibrinopeptide A and its analogs may play a protective role, while COL1A1-OxP and DVP represent potential new markers of bone turnover. These findings suggest metabolomics-based biomarkers could aid clinical decision-making by allowing earlier detection of rebound effects and guiding individualized treatment strategies after denosumab therapy.

**Clinical trial registration number:** ClinicalTrials.gov, NCT01630941 (URL: https://clinicaltrials.gov/); European Union Clinical Trials Register (EU CTR), EudraCT No. 2011-001481-18 (https://www.clinicaltrialsregister.eu/)

## Graphical Abstract

**Graphical Abstract:**
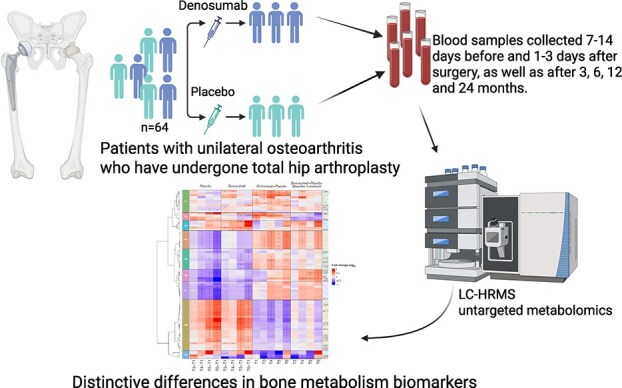

## Introduction

Bone turnover is a lifelong process governed by a finely tuned balance between bone formation and resorption, maintained by the coordinated activity of osteoblasts and osteoclasts. Disruption of this balance can lead to skeletal disorders such as osteoporosis, a leading cause of fractures and mortality, particularly among postmenopausal women. A key player in osteoclast differentiation and activation is the RANKL, which binds to its sole receptor, RANK, on osteoclast precursors. The essential role of this signaling axis is underscored by the complete absence of osteoclastogenesis in RANK-deficient mice.^1^

Denosumab is a fully human monoclonal antibody targeting RANKL, inhibiting osteoclast maturation and survival.^2^ Its clinical use in patients with osteoporosis has been shown to increase BMD and reduce the risk of vertebral, nonvertebral, and hip fractures.^3^^,^^4^ However, upon treatment cessation, patients often experience a rebound effect characterized by a sharp increase in biochemical markers of bone resorption and rapid loss of previously gained BMD and fracture protection.^5–7^

Total hip arthroplasty (THA) is widely regarded as one of the most successful orthopedic procedures, due to its consistently high rates of patient satisfaction, substantial improvements in pain relief and functional mobility, and excellent long-term implant survival. Nonetheless, implant loosening, partially driven by local osteoclast-mediated bone resorption, is the most common reason for revision after THA, and thus remains a significant clinical concern.^8^ Periprosthetic bone loss around cemented and uncemented femoral stems is a well-documented phenomenon, often attributed to osteoclast activation mainly due to stress shielding in the bone around the implant.^9^^,^^10^ Various attempts to mitigate this bone loss using bisphosphonates have largely failed to produce consistent results.^11–13^ Until recently, the role of denosumab in this context had not been explored.

To address this gap, we initiated a randomized, double-blind, placebo-controlled clinical trial (RCT) to evaluate the effects of denosumab on periprosthetic bone loss in patients undergoing uncemented THA.^9^ The study enrolled middle-aged patients (35-65 yr) with unilateral osteoarthritis of the hip (OAH) and no evidence of osteoporosis. Participants were randomized to receive 2 doses of denosumab or placebo, administered postoperatively. The results demonstrated that denosumab significantly prevented early periprosthetic bone loss. However, this protective effect diminished after treatment discontinuation, and notably, serum concentrations of RANKL were elevated at 6 and 12 mo postoperatively in the denosumab group,^14^ suggesting that the rebound phenomenon may be mediated by enhanced osteoclast activity.

Understanding the biological mechanisms underpinning this rebound effect is critical, not only within the scope of this trial but also for the broader population receiving denosumab for osteoporosis. Advances in omics technologies, particularly metabolomics, offer powerful tools for uncovering molecular signatures associated with disease and treatment response. Metabolomics has emerged as a promising approach in the study of osteoarthritis and bone metabolism,^15^^,^^16^ enabling the comprehensive profiling of small molecules and peptides that reflect underlying physiological states.

To investigate the molecular mechanisms involved in the rebound effect observed after denosumab discontinuation, we applied untargeted high-resolution mass spectrometry coupled with liquid chromatography to analyze serum samples from patients enrolled in our original randomized controlled trial. This study aimed to identify metabolic and peptidomic alterations associated with denosumab treatment and its withdrawal, and it also focused on identifying potential biomarkers of bone turnover. If validated, such biomarkers could be incorporated into clinical practice to support early detection of rebound activity and guide decisions on sequential therapies or individualized monitoring after treatment cessation.

## Methods

### Trial design

A prospective, randomized, double-blind, placebo-controlled phase 2 clinical trial was performed in patients with unilateral OAH who underwent cementless THA to investigate whether a RANKL-inhibitor could inhibit periprosthetic BMD loss that regularly occurs after such interventions.^17^ Sixty-four patients were randomized to either 2 subcutaneous doses of denosumab (*n* = 32) or placebo (*n* = 32) given 1-3 d and 6 mo after surgery. Patients were followed for 24 mo. Morning fasting blood samples were collected 7-14 d before surgery (T1), 1-3 d (T2) and 3 (T3), 6 (T4), 12 (T5), and 24 (T6) months after surgery, resulting in 6 sequential samples from each patient. The trial’s primary and selected secondary outcomes have been described in detail previously.^9^

### Ethics approval

The trial was registered at ClinicalTrials.gov 2011-001481-18, NCT01630941, and approved by the Regional Ethics Committee in Uppsala, Sweden (2011/297/3).

### Subjects

All patients (*n* = 461) aged 35-65 yr, referred to the Department of Orthopaedics, Uppsala University Hospital, were assessed for eligibility between August 7, 2012 and January 21, 2015. Inclusion criteria were radiologically verified OAH defined as Kellgren–Lawrence (KL) grades 3-4 of the affected hip. Exclusion criteria were contralateral OAH above KL grade 1 or previous THA, bodyweight >110 kg or BMI >35 kg/m^2^, treatment with bone-modulating drugs or corticosteroids, malignancy, American Society of Anesthesiologists (ASA) class >3, substance abuse, pregnancy, previous exposure to large doses of irradiation, or otherwise unsuitable as judged by the investigators.

### Randomization and blinding

At the start of the study, 64 patients were eligible. They started a daily treatment of calcium (500 mg) and vitamin D3 (800IE) 7-14 d before the procedure and up to 1 yr after. The sealed envelope technique was used to randomize patients to receive an injection of either 60 mg of denosumab or a placebo (0.9% saline), for details see the study by Nyström et al. (2020).^9^

### Peri- and postoperative procedures and implants

One of the 2 surgeons performed cementless THA using a Continuum cup with a highly cross-linked polyethylene-elevated liner, a collum femoris-preserving stem, and a 28-mm CoCr head. The subcutaneous denosumab injections or placebo were given after baseline DXA had been performed. Fasting morning blood samples were drawn 1-3 d postoperatively, prior to the first administration of denosumab. The second injection of denosumab was given 6 mo later.

### Biochemical markers of bone metabolism

In total, 1-2 mL of morning fasting blood samples were collected, serum prepared, and stored at −70 °C. Carboxy-terminal telopeptide of type 1 collagen (CTX, β-CrossLaps, Cobas, Roche) was measured as a marker of bone resorption and procollagen type 1 amino-terminal propeptide (P1NP, Cobas, Roche) was quantified as a reference for bone formation.^18^ The assays were performed in a laboratory certified in accordance with ISO 15189:201, and the coefficient of variation was 3% for P1NP and 6% for CTX.

### Metabolite extraction

Serum samples were carefully thawed on ice to preserve metabolite integrity before undergoing protein precipitation for metabolite extraction. This was achieved using a precipitation solution consisting of methanol (LC-MS grade, from Honeywell), which was presupplemented with internal standards to support downstream quantification and normalization. For each sample, 50 μL of serum was mixed with 150 μL of ice-cold precipitation solution in a 1.5 mL Eppendorf tube. The mixture was vortexed for 15 s to ensure thorough mixing and then incubated at −20 °C for 60 min to enhance protein precipitation.

Following incubation, the samples were centrifuged at 19 300 g for 10 min at 4 °C. This process resulted in a clear supernatant, from which 100 μL was carefully transferred to a HPLC glass vial for analysis.

Samples were processed in preparation batches, each containing up to 23 individual samples. A batch-specific pool was created to monitor technical variability within each batch by combining 10 μL aliquots from each prepared sample. These pooled batch samples were stored at −80 °C until analysis. After all batches were processed, the individual batch pools were combined to form a single grand pool, representing all samples included in the study. This grand pool was also stored at −80 °C and served as a standardized quality control (QC) sample throughout all analytical experiments to monitor instrument performance and ensure data consistency.

### Sample injection and LC-HRMS analysis

The biological samples were analyzed using a structured injection sequence designed to ensure analytical consistency and minimize technical variability. Specifically, the samples were injected in a constrained randomized order to avoid bias due to instrument drift or systematic effects. A QC sample was injected after every 10th to 12th sample to monitor instrument stability and performance. Each QC injection was immediately followed by a blank injection to assess potential carryover and contamination.

The chromatographic column was preconditioned before injecting the study samples to stabilize its performance. This preconditioning involved 5 repeated QC sample injections followed by 2 blank injections to ensure a clean and consistent baseline.

For each analysis, 2 μL of the prepared sample was injected onto a reversed-phase HPLC column (Accucore aQ C18, 100 × 2.1 mm, 2.6 μm particle size, Thermo Scientific). The separation was conducted using an Ultimate 3000 HPLC system (Thermo Scientific), which was coupled to a high-resolution hybrid quadrupole Orbitrap mass spectrometer (Q Exactive Orbitrap, Thermo Scientific) to detect and identify metabolites. The samples were separated using gradient elution with mobile phase A consisting of MQ-water and 0.1% formic acid (American Chemical Society reagent grade, from Merck), and mobile phase B consisted of 90% acetonitrile (LC-MS grade, VWR Chemicals), and 10% isopropanol (LC-MS grade, VWR Chemicals) with 0.1% formic acid. MQ-water was purified using an Advantage A10 Milli-Q system (Merck Millipore). All samples were analyzed using full scan mode under positive ionization conditions, allowing for a broad detection range of positively charged metabolites. In addition to this, more detailed structural information was obtained through tandem mass spectrometry (MS/MS) analyses. These MS/MS experiments were carried out on pooled subsets of the samples using 3 different levels of normalized collision energy (NCE), specifically NCE = 20, 30, and 40, to maximize fragmentation coverage. Comprehensive technical details regarding the liquid chromatography–high-resolution mass spectrometry (LC-HRMS) setup and conditions have been published previously.^19^

### Quantification and normalization

The acquired raw data from the metabolomics analysis was first converted into an open-source format (.mzML) for compatibility with various bioinformatics tools. This conversion and centroiding (a process that simplifies spectral data by focusing on peak centers) were carried out using the “msconvert” utility from the ProteoWizard software suite.^20^ The preprocessed data were then analyzed using a pipeline built within the KNIME analytical platform,^21^ employing modules from the OpenMS metabolomics toolkit.^22^

Following centroiding, the data underwent several key preprocessing steps. First, metabolite features were quantified using the “FeatureFinderMetabo” tool. Next, the alignment of features across all samples was performed using “MapAlignerPoseClustering,” which corrects for slight variations in retention time. After alignment, features were linked across different samples using “FeatureLinkerUnlabelledQT,” ensuring that corresponding metabolites were grouped correctly. Within this step, a time tolerance of 10 s and a mass deviation tolerance of 5 ppm (ppm) were applied to enhance feature-matching accuracy.

The resulting dataset, containing quantified metabolite features, was imported into the statistical computing environment R (version 3.6.0; R Core Team, 2019) for further processing. To ensure data quality and relevance, contaminants were filtered out by referring to the blank injection runs, following a pipeline we previously established.^23^ In addition, only those metabolic features that showed a statistically significant Pearson correlation (*p*-value < .05) with a dilution series were retained, further ensuring the robustness of the data.

To prepare the intensity data for statistical analysis and stabilize variance, all values were log_2_-transformed. Finally, a LOESS normalization was applied to correct for systematic variation due to run-order effects, such as signal intensity decay over time. Specifically, LOESS curves were fitted for each metabolite using the “loessFit” function from the R package limma, with a smoothing parameter (span) of 0.2 used to adjust the intensity trends across the run.^24^

### Statistical analyze

Before conducting statistical analyses, we performed data filtering to ensure reliability. Features (such as metabolite measurements) with less than 80% data coverage, meaning more than 20% of their values were missing across all samples, were excluded. In addition, we removed features with missing values at the presurgery time point (T1) for any individual. This step ensured that baseline correction could be applied consistently across all samples and features.

We used multilevel principal component analysis (PCA) to handle the remaining missing data. This technique was implemented via the NIPALS algorithm using the R package mixOmics,^25^ allowing for the imputation of missing values during analysis.

Subsequently, we analyzed each metabolite individually using linear mixed-effects models, which are well-suited for datasets involving repeated measures from the same subjects. This modeling was performed with the R package lme4.^26^ In these models, the metabolite concentration served as the dependent variable. The independent variables included fixed effects for age, sex, BMI, experimental plate (to account for technical variability), visit (time point), treatment group, and the interaction between visit and treatment. Patient identity was included as a random effect to account for individual variability.

We used likelihood ratio tests to compare models with and without these variables to assess the overall influence of visits and treatment. These analyses were conducted on both raw metabolite levels and baseline-corrected levels, where the correction was based on individual T1 values. To determine statistical significance, a *p*-value threshold of <.0001 was applied. This threshold corresponds to a family-wise error rate of 0.01 and was derived using a less conservative version of the Bonferroni correction, assuming roughly 100 independent tests.

The same modeling approach was also applied to the scores from the first 20 principal components (PCs) from the PCA and to levels of 2 bone metabolism biomarkers, SP1NP and CTX, which reflect bone formation and resorption, respectively.

To further explore differences, we estimated marginal means and performed post hoc pairwise comparisons using Tukey’s test, implemented with the R package means (version 1.7.0). These comparisons were used to identify significant differences between the denosumab and placebo groups at each time point and to examine within-group changes over time. In these post hoc analyses, a *p*-value of <.05 was considered statistically significant.

We also analyzed the association between the intensities of selected significant metabolites and the 2 bone biomarkers. These associations were examined using linear models, adjusting for the treatment group, sex, BMI, and age to control for potential confounding variables.

Finally, to visualize metabolic patterns, we conducted hierarchical clustering based on the log_2_ fold-changes of metabolites that showed significant effects. This clustering was performed using the stats package in R, applying Ward’s method, which organizes metabolites into clusters that reflect similarities in their response patterns.

### Metabolite and peptide identification

To annotate the detected metabolic features, we applied 2 complementary identification strategies using the MetaboIGNITER workflow (https://nf-co.re/metaboigniter). The first approach was an in-house identification method. Here, metabolite identities were confirmed by comparing the detected features to an internal library containing 471 characterized pure reference standards. These standards had been analyzed using the exact same LC-HRMS conditions as those applied to the study samples. A metabolite was considered positively identified if both its retention time (within a 5-s deviation) and its mass deviation (within 5 ppm) matched a reference feature in the in-house library.

The second approach involved in silico identification, which was carried out using 2 distinct methods. First, all detected features were queried against public compound databases using the SIRIUS platform,^27^ a widely used tool for molecular formula and structure prediction based on tandem MS data. Here, we used a maximum precursor mass deviation of 5 parts per million (ppm) and a fragment mass deviation of 20 ppm, ensuring high precision in spectral matching.

In addition to small molecule identification, we also searched for endogenous peptides using the X!Tandem search engine.^28^ This analysis was performed with unspecific enzymatic cleavage settings to allow for broad peptide detection. The search was conducted against an in-house protein database containing all known human precursor proteins, as previously described in our earlier work.^29^ A preprecursor mass deviation of 5 ppm and a fragment mass tolerance of 0.03 Da were used for peptide identification, along with a 5-s retention time deviation criterion. These procedures’ metabolite and peptide identifications were carefully and manually curated to ensure accuracy and biological relevance.

## Results

After filtering the dataset to exclude features with inadequate coverage or missing baseline values, 1552 metabolite features remained for downstream analysis. A multilevel PCA was performed to explore overall trends in the dataset. This unsupervised analysis revealed a pronounced shift in the metabolomic profile 1-3 d after surgery (T2), as illustrated in Figure S1.

To determine the number of metabolite features significantly associated with either time point, treatment, or both, including the immediate postsurgical time point (T2), linear mixed-effects models were fitted for each feature. This analysis identified 1145 significantly altered features, with a *p*-value threshold of <.0001. This corresponds to approximately 74% of all analyzed metabolites, indicating a widespread metabolic response shortly after surgery. However, when the immediate postsurgical time point (T2) was excluded from the analysis, significantly altered features dropped to 83, suggesting that the acute response following surgery accounted for most changes. Among these 83 features, 79 overlapped with those initially found to be significant when T2 was included.

Two additional linear mixed-effects models were constructed to better isolate the effect of time after surgery, including the time point variable and one without it. These models were then compared using a likelihood ratio test. This comparison identified 75 features as significantly associated with time after surgery, implying that these metabolites exhibited sustained changes beyond the immediate postoperative period. This result indicates that approximately 6.5% of the measured metabolite features continued to be affected over the longer-term following surgery.

**Figure 1:**
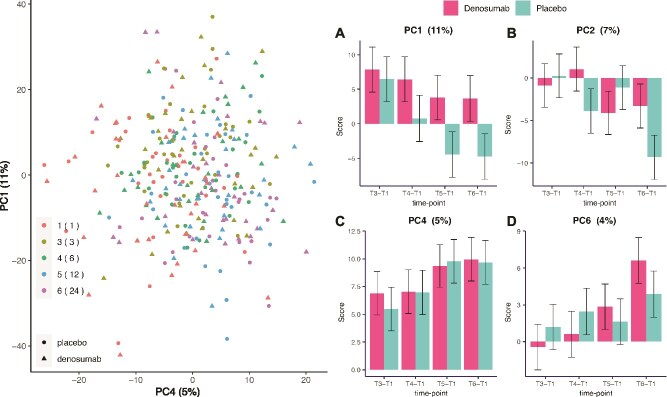
PCA plot based on 1552 metabolites and all time points except T2. Left: PCA plot based on 1552 metabolites (excluding T2) illustrates the distribution of samples over time, with colors indicating time points and shapes representing treatment groups (denosumab or placebo). Right: subplots (A–D) show selected PCs (PC1, PC2, PC4, and PC6) that revealed significant differences between time points in each treatment group relative to baseline (T1). Asterisks denote statistically significant differences (*p* < 0.05), and error bars represent standard errors. Time points: T1 (baseline), T3 (3 mo), T4 (6 mo), T5 (12 mo), and T6 (24 mo). The second dose of denosumab was administered at 6 mo.

To focus the analysis on the intermediate and long-term effects of the therapeutic intervention, the T2 time point was excluded from subsequent investigations. A new PCA was conducted on the filtered dataset, and to assist in interpretation, linear mixed-effects models were also fitted to the resulting PCA scores. Ten PCs were found to be significantly associated with either time point or treatment (*p* < .05), together explaining 36.1% of the total variance in the dataset. Among these, PC1 explained 11%, PC2 explained 7%, PC4 explained 5%, and PC6 explained 4% of the variance. The most prominent differences across components were attributed to changes between time points, as shown in Figure 1 and Table S1. PC4 captured significant changes in the metabolome after surgery, which were observed similarly in both the denosumab and placebo groups. In contrast, PC1, PC2, and PC6 reflected metabolic differences modulated differently between the treatment groups, highlighting a treatment-specific response to denosumab over time.

To further dissect which specific features were significantly influenced by time and/or treatment, linear mixed-effects models were again applied to each feature, excluding T2. Eighty-three features were identified as statistically significantly altered (*p* < .0001). For these features, within-time point contrasts between the denosumab and placebo groups were calculated, both with and without correction for baseline differences, and the results are presented in Table S2.

**Figure 2:**
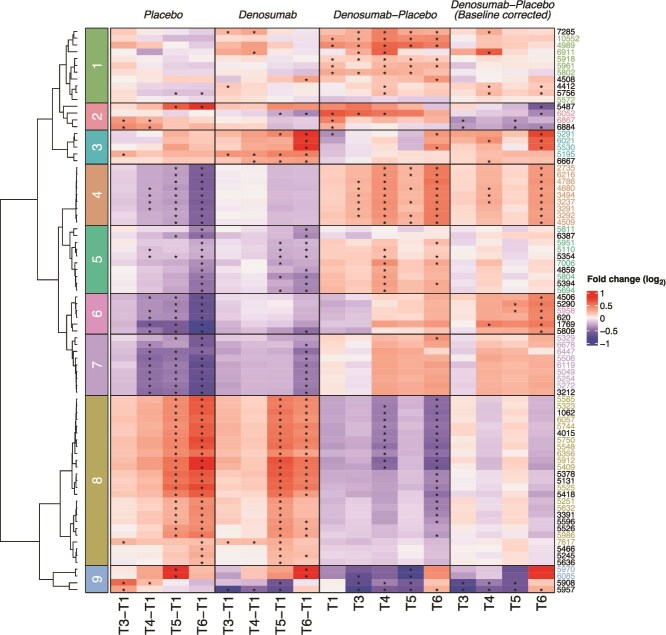
Hierarchical clustering of the significantly affected metabolites. Heatmap displaying log_2_ fold changes of 83 significantly affected metabolites, stratified by time and treatment comparisons. Clustering highlights metabolite dynamics within placebo and denosumab groups over time and between-group comparisons (denosumab vs placebo), with and without baseline correction. Asterisks indicate statistical significance (*p* < 0.05). Metabolite IDs correspond to Table 1 for compound identity and class. Time points: T1 (baseline), T3 (3 mo), T4 (6 mo), T5 (12 mo), and T6 (24 mo). The second dose of denosumab was administered at 6 mo.

To facilitate visualization and pattern recognition, hierarchical clustering was performed on the log_2_-transformed pairwise fold changes of these significant features (Figure 2). This analysis revealed 9 distinct clusters, each representing shared dynamic patterns over time or between treatments. These clusters corresponded to 30 identified compounds, including 27 unique metabolites, lipids, and amino acid sequences, as summarized in Table 1.

**Table 1:** The identified features with corresponding codes are referred to in Figure 2.

**Cluster**	**Code**	**ANOVA (*p*-value)**	**Description/Name**	***m*/*z***	**Class**	**Chemical composition/Amino acid sequence**	**Adduct**
**1**	7285	2.41E−06	Phosphatidylserine(O-18:0/0:0), synonym LPS(O-18:0)	534.3175	Glycerophospholipid	C24H50NO8P	[*M* + Na]+
**1**	4508	1.97E−05	Tiglylcarnitine	244.1551	Metabolite	C12H21NO4	[*M* + H]+
**1**	4412	9.03E−05	Lyso-PAF C-16, synonym LPC(o16:0)	482.3614	Glycerophospholipid	C24H52NO6P	[*M* + H]+
**1**	5756	4.20E−06	LysoPC(16:0/0:0), synonym LPC(16:0)	504.3434	Glycerophospholipid	C24H50NO7P	[*M* + Na]+
**2**	5487	2.10E−08	Fibrinogen beta chain	565.2271	Peptide	NDNEEGFFSA	[*M* + 2H]+
**2**	6884	7.52E−05	4-Hydroxyproline	132.0657	Amino acid	C5H9NO3	[*M* + H]+
**3**	6667	1.09E−05	Phenylalanine	188.0683	Amino acid	C9H11NO2	[*M* + Na]+
**5**	6387	1.00E−05	Alpha-glutamylglycylphenylalanine	352.1515	Tripeptide	EGF	[*M* + H]+
**5**	5354	7.21E−09	Complement C4B (Chido blood group)	793.4106	Peptide	DDPDAPLQPVTPLQLFEGRRN	[*M* + 3H]+
**5**	4859	4.24E−06	Fibrinogen alpha chain	749.384	Peptide	FLAEGGGV	[*M* + H]+
**5**	5394	6.55E−07	Fibrinogen alpha chain	375.1958	Peptide	FLAEGGGV	[*M* + 2H]+
**6**	5290	4.81E−05	Di-L-phenylalanine	335.1374	Dipeptide	FF	[*M* + Na]+
**6**	620	5.19E−06	Di-L-phenylalanine	313.1553	Dipeptide	FF	[*M* + H]+
**6**	1769	5.55E−05	Phenylalanylleucine	279.1712	Dipeptide	FL	[*M* + H]+
**6**	5809	1.44E−08	Alpha-Asp-Phe	281.1136	Dipeptide	DF	[*M* + H]+
**6**	4506	3.19E−06	Phenylalanine (in source fragment of FF)	166.0866	Amino acid (in source fragment)	F	[*M* + H]+
**7**	3212	4.11E−06	Fibrinogen beta chain	472.6847	Peptide	Q(Ammonia-loss)GVNDNEEG	[*M* + 2H]+
**8**	1062	1.45E−08	Fibrinogen alpha chain	733.3333	Peptide	DSGEGDFLAEGGGVR	[*M* + 2H]+
**8**	4015	7.39E−09	Fibrinogen alpha chain	489.2244	Peptide	DSGEGDFLAEGGGVR	[*M* + 3H]+
**8**	5378	1.12E−10	Fibrinogen alpha chain	450.882	Peptide	SGEGDFLAEGGGVR	[*M* + 3H]+
**8**	5131	1.20E−10	Fibrinogen alpha chain	675.8197	Peptide	SGEGDFLAEGGGVR	[*M* + 2H]+
**8**	5418	1.10E−09	Fibrinogen alpha chain	632.3035	Peptide	GEGDFLAEGGGVR	[*M* + 2H]+
**8**	3391	2.53E−06	Fibrinogen alpha chain	603.7924	Peptide	EGDFLAEGGGVR	[*M* + 2H]+
**8**	5596	1.45E−06	Fibrinogen alpha chain	402.8639	Peptide	EGDFLAEGGGVR	[*M* + 3H]+
**8**	5526	1.46E−08	Fibrinogen alpha chain	539.2712	Peptide	GDFLAEGGGVR	[*M* + 2H]+
**8**	5466	2.55E−05	Fibrinopeptide A	768.852	Peptide	ADSGEGDFLAEGGGVR	[*M* + 2H]+
**8**	5245	5.06E−06	Fibrinopeptide A	512.9037	Peptide	ADSGEGDFLAEGGGVR	[*M* + 3H]+
**8**	5636	1.53E−05	PDZ domain containing 7	759.8471	Peptide	ADTAMQTEPDAGGRV	[*M* + 2H]+
**9**	5908	1.01E−34	Collagen type I alpha 1 chain, named COL1A1-OxP	626.2856	Peptide	AP(Ox)GDRGEP(Ox)GPP(Ox)GP	[*M* + 2H]+
**9**	5957	1.74E−49	DL-alpha-aspartyl-DL-valyl-DL-proline	330.1669	Peptide	C14H23N3O6 / (DVP)	[*M* + H]+

**Figure 3:**
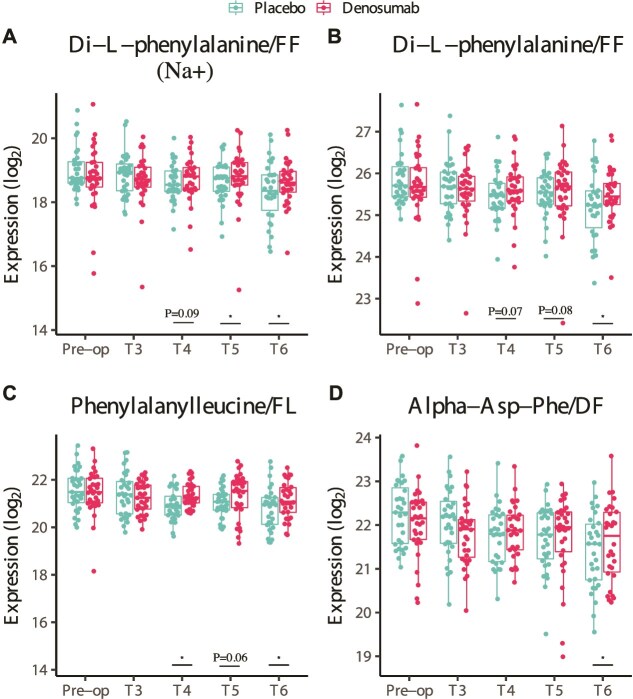
Temporal changes in serum dipeptide levels following surgery. Boxplots showing serum concentrations of 4 dipeptides: (A, B) Di-L-phenylalanine (including sodium adduct), (C) phenylalanylleucine, and (D) alpha-Asp-Phe. In denosumab-treated patients, all dipeptides increased significantly over time, starting at 6 mo postoperatively. Asterisks mark statistically significant differences (*p* < 0.05); exact *p*-values are indicated when *p* < 0.1. Time points: T1 (baseline), T3 (3 mo), T4 (6 mo), T5 (12 mo), and T6 (24 mo). The second dose of denosumab was administered at 6 mo.

Following surgery, several dipeptides exhibited distinct concentration patterns between treatment groups. In placebo-treated patients, there was a significant decrease in the serum levels of the dipeptides di-L-phenylalanine, phenylalanylleucine, and alpha-Asp-Phe (*p* < .05). This decline, categorized under cluster 6, was not observed in the denosumab-treated group. Notably, in the denosumab group, the concentrations of all 3 dipeptides began to increase from 6 mo post-surgery. They continued to rise at 12 and 24 mo, as illustrated in Figure 3.

In contrast to these trends, the bone turnover marker 4-hydroxyproline (cluster 2) displayed significantly decreased serum levels 3, 12, and 24 mo after surgery, indicating a consistent reduction in bone resorption over time in both the intervention and the control group. Similarly, the peptide NDNEEGFFSA, derived from the fibrinogen beta chain (FIBB), also exhibited significantly decreased levels at 24 mo post-surgery and was classified within the same cluster as 4-hydroxyproline.

Cluster 9 comprised 4 features reflecting long-term treatment-specific effects between the denosumab and placebo groups. Two of these features were identified as the oxidized peptide AP(Ox)GDRGEP(Ox)GPP(Ox)GP, referred to as COL1A1-OxP, derived from the collagen type I alpha one chain (COL1A1). Another compound in this cluster, with the chemical formula C14H23N3O6, was identified as the tripeptide DL-alpha-aspartyl-DL-valyl-DL-proline (DVP).

**Figure 4:**
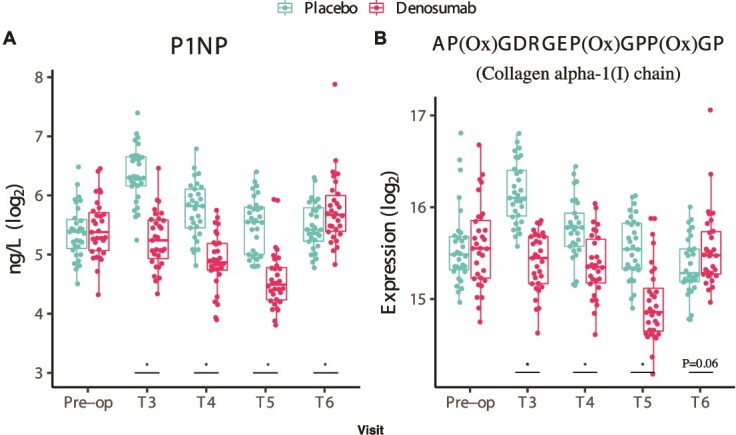
Trends in bone formation marker P1NP and COL1A1-OxP peptide. Boxplots depicting serum levels of P1NP and the oxidized peptide AP(ox)GDRGEP(ox)GPP(ox)GP (COL1A1-OxP), derived from the collagen type I alpha 1 chain. Both markers showed sustained suppression in denosumab-treated patients up to 12 mo, with a trend toward rebound by 24 mo. Asterisks denote *p* < 0.05; *p* < 0.1 values are shown explicitly. Time points: T1 (baseline), T3 (3 mo), T4 (6 mo), T5 (12 mo), and T6 (24 mo). The second dose of denosumab was administered at 6 mo.

The serum levels of COL1A1-OxP were significantly lower in denosumab-treated patients at 3-, 6-, and 12-mo post-surgery. At 24 mo, a slight, nonsignificant increase was observed in the denosumab group compared to placebo (*p* = .06). Interestingly, this trajectory mirrored the expression profile of the bone formation marker P1NP, which also showed a late increase at 24 mo. A strong correlation between COL1A1-OxP and P1NP was observed (*p* = 4.4E^−83^), as shown in Figure 4.

**Figure 5:**
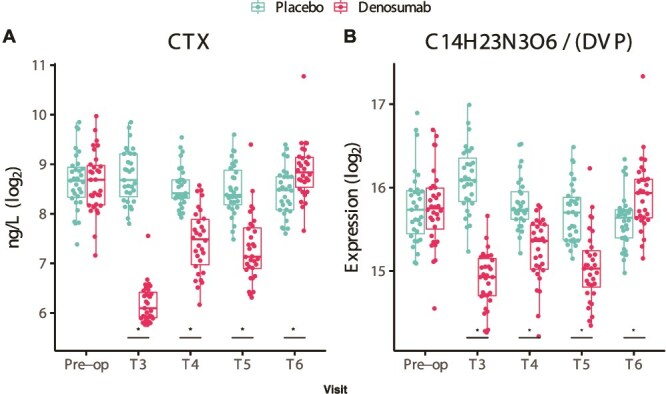
Trends in bone resorption marker CTX and tripeptide DVP. Boxplots illustrating serum levels of CTX (a marker of bone resorption) and DL-alpha-aspartyl-DL-valyl-DL-proline (DVP), a newly identified tripeptide. Both markers remained suppressed in the denosumab group until 12 mo, followed by a notable increase at 24 mo. Asterisks represent statistically significant changes (*p* < 0.05), and exact *p*-values are noted for trends (*p* < 0.1). Time points: T1 (baseline), T3 (3 mo), T4 (6 mo), T5 (12 mo), and T6 (24 mo). The second dose of denosumab was administered at 6 mo.

A similar pattern was noted for DVP. Its concentration was lower in denosumab-treated patients for up to 12 mo following surgery, followed by a significant increase at 24 mo. This increase closely resembled the bone resorption marker CTX pattern, with DVP showing a highly significant correlation with CTX (*p* = 1.1E^−222^), as illustrated in Figure 5.

Cluster 8 consisted predominantly of peptides derived from the fibrinogen alpha chain (FIBA), including the well-characterized fibrinopeptide A (ADSGEGDFLAEGGGVR). These peptides demonstrated significantly elevated concentrations at 12 and 24 mo after surgery. At 24 mo, most of these peptides, fibrinopeptide A included, showed statistically significant differences between the denosumab and placebo groups (*p* < .05). However, no significant differences were observed between the groups at baseline. When baseline values were considered, the differences in peptide concentrations between treatment groups at 24 mo were no longer statistically significant, suggesting that treatment effects were conditional on baseline expression.

Among the few lipid-related metabolites, the glycerophospholipid Lyso-PAF C-16, also known as LPS(O-18:0) and LysoPC(16:0/0:0) (synonym LPC(16:0)), showed a significant increase in concentration 24 mo after surgery in denosumab-treated patients (cluster 1). Finally, the metabolomic features grouped within clusters 3, 4, and 7 could not be identified with high confidence despite rigorous efforts and thus remain unannotated in the current analysis.

## Discussion

In this post hoc analysis of patients enrolled in a previous randomized controlled trial investigating the effects of denosumab treatment on periprosthetic BMD, we observed a pronounced shift in the serum metabolome occurring 1-3 d after surgery. This acute response affected approximately 74% of all measured metabolite features. Furthermore, about 6.5% of the metabolite features remained altered over the longer term, suggesting some sustained metabolic change following surgery.

While these findings indicate a robust metabolic response to surgical intervention, interpretation of the immediate postsurgical effects requires caution. Our study did not include a control group that avoided surgery or was free from unilateral hip osteoarthritis. As a result, we believe it is plausible that the observed changes shortly after surgery are attributable to the surgical trauma itself. However, we cannot exclude that the underlying osteoarthritic condition or age-related metabolic shifts play confounding roles. Therefore, the widespread metabolic changes seen at the immediate postsurgical time point (T2) may be confounded by factors unrelated to the intervention. To minimize this uncertainty and better isolate denosumab’s intermediate and long-term metabolic effects, we excluded the T2 time point from subsequent analyses.

Beyond the immediate postsurgical period, we identified several treatment-specific metabolic changes. Notably, the dipeptides di-L-phenylalanine, phenylalanylleucine, and alpha-Asp-Phe increased in denosumab-treated patients but not in those receiving a placebo. In contrast, peptides derived from the FIBA showed rising concentrations in both groups over time. In addition, we identified 2 novel peptides, AP(Ox)GDRGEP(Ox)GPP(Ox)GP, derived from the collagen type I alpha one chain (COL1A1, here referred to as COL1A1-OxP), and the tripeptide DL-alpha-aspartyl-DL-valyl-DL-proline (DVP). Both peptides were strongly correlated with ELISA-based CTX measurements, a bone resorption marker, and P1NP, a marker of bone formation.

We have previously shown that denosumab effectively prevents early periprosthetic bone loss following uncemented THA, but that this effect is followed by a considerable rebound after treatment discontinuation.^9^ This is consistent with previous reports showing increased biochemical markers of bone metabolism, reversal of BMD gains, and loss of vertebral fracture protection after denosumab cessation.^4^^,^^30^^,^^31^ Supporting the hypothesis that RANKL mediates the reactivation of dormant osteoclasts, we also found elevated serum RANKL concentrations at 6 and 12 mo postoperatively in denosumab-treated patients.^14^

To further explore the rebound effect following denosumab withdrawal after uncemented THA, we employed metabolomics technologies to examine the global impact of the original RCT on the serum metabolome, where patients had been randomized to receive either denosumab or placebo.^9^ Surgery induced similar changes in serum metabolomics in both groups initially, but the overall metabolomic profiles diverged by 6 mo. Interestingly, these changes were primarily attributed to endogenous peptides rather than classical metabolites or lipids. Upon deeper investigation, we observed that levels of Di-L-phenylalanine (also known as phenylalanylphenylalanine), phenylalanylleucine, and alpha-Asp-Phe (also known as aspartyl-phenylalanine) decreased significantly in placebo-treated patients after surgery but remained stable or increased in denosumab-treated patients.

Dipeptides are biologically intriguing molecules with proposed antioxidant properties^32^ and associations with pathological processes, including cancer. Increased serum levels of phenylalanylphenylalanine, phenylalanylleucine, and aspartyl-phenylalanine have been linked to an elevated risk of pancreatic ductal adenocarcinoma.^33^ In addition, high serum phenylalanine levels have been associated with the progression of knee osteoarthritis in women,^34^ and aspartyl-phenylalanine concentrations have been positively correlated with disease activity in rheumatoid arthritis.^35^ All dipeptides in our study contained phenylalanine and showed significantly higher concentrations in denosumab-treated patients. For phenylalanylleucine, this difference emerged at 6 mo and continued to increase through 24 mo. These findings suggest a potential role for dipeptides in the mechanisms driving rebound bone loss after denosumab withdrawal. No significant sex-based differences in dipeptide concentrations were observed, though sample size limitations must be acknowledged.

While an inflammatory component in OAH has been proposed,^36^ our recent analysis of 74 cytokines and inflammation-related markers showed no significant effects of denosumab treatment on this group.^14^ Amino acids play key roles in immune responses, including regulating immunoglobulins and cytokines.^37^ In a study by Stolzenberg-Solomon et al., the fibrinogen alpha peptide DSGEGDFXAEGGGVR was associated with a reduced incidence of pancreatic ductal adenocarcinoma.^33^ We identified 6 unique peptides derived from the N-terminal region of fibrinogen alpha, including the intact form of fibrinopeptide A (ADSGEGDFLAEGGGVR). These peptides increased over time in both treatment arms. However, concentrations were significantly higher in placebo-treated patients 6 mo after surgery. The same trend reappeared at 24 mo, 18 mo after the second denosumab dose, where levels were again significantly higher in the placebo group, although without baseline adjustment. Fibrinogen is crucial to the terminal steps of the coagulation cascade, and fibrinopeptide A exists in both phosphorylated and unphosphorylated forms. Its phosphorylation increases substantially during acute-phase reactions, with up to 60% of the segment potentially phosphorylated.^38^ We identified and matched the phosphorylated serine-containing form but found no significant treatment-specific differences. The link between fibrinogen alpha peptides and the rebound effect following denosumab withdrawal has not previously been reported but may be relevant for understanding the associated changes in BMD.

We have previously demonstrated that the bone metabolism markers P1NP and CTX are suppressed in the denosumab group up to 12 mo after surgery. At 24 mo, however, both markers were elevated in this group, coinciding with reduced BMD differences between denosumab and placebo.^9^ Interestingly, 4-hydroxyproline showed decreased levels 24 mo after surgery, supporting the notion that its serum concentration may serve as a nonspecific indicator of collagen turnover.

Among our most significant findings were 2 novel peptides strongly correlated with bone metabolism markers. The peptide COL1A1-OxP is derived from the collagen type I alpha one chain (COL1A1), spanning amino acids 798-810 and including three 4-hydroxyproline residues at positions 799, 805, and 808. Together with COL1A2, COL1A1, a 139 kDa protein comprising 1464 amino acids, is the main structural protein in the bone matrix. During bone formation, it is cleaved to yield N-terminal P1NP and C-terminal PICP fragments. P1NP, cleaved from amino acids 23-161, is a 35 kDa circulating protein in intact trimeric and monomeric forms.^39^ As P1NP is released during new collagen synthesis, it reflects bone formation, similar to how C-peptide reflects insulin production. Although type I collagen is also present in soft tissues such as skin and muscle, bone turnover is typically faster, making procollagen changes most representative of bone activity.^39^ The ELISA assay used here (Roche Diagnostics) measures total P1NP, but the intact form appears more clinically informative.^39^ The newly identified peptide, located at amino acids 798-810, falls within the mature region of collagen (amino acids 162-1218), indicating it is more likely a marker of bone degradation. Despite this, COL1A1-OxP showed strong correlations with P1NP (*p* = 4.4E^−83^) and CTX (*p* = 1.5E^−53^), with the strongest association with P1NP at 3 mo post-surgery. This time point is also when P1NP and CTX diverge most, suggesting COL1A1-OxP may represent a novel marker of bone formation.

We also identified DVP as a potential novel marker of bone resorption. DL-alpha-aspartyl-DL-valyl-DL-proline (DVP) correlated strongly with CTX (*p* = 1.1E^−222^) and P1NP (*p* = 9.3E^−23^), although the correlation with CTX was substantially stronger. As with COL1A1-OxP, the time point at 3 mo post-surgery distinguished its relationship to bone turnover dynamics. Cross-linking stabilizes newly deposited collagen fibrils; the primary cross-links in bone are pyridinoline and deoxypyridinoline.^40^ Serum CTX reflects degradation of the C-terminal telopeptide of type I collagen and is generated via cathepsin K cleavage, with the sandwich assay recognizing the EKAHDGGR sequence (amino acids 1207-1214). The strong correlation between DVP and CTX suggests that DVP may also be released during collagen breakdown.

Carboxy-terminal telopeptide of type 1 collagen (CTX) has been proposed as a guide for initiating sequential therapies after denosumab withdrawal.^41^ However, newer studies suggest that relying on elevated CTX may be too late, as bone loss may have already occurred.^42^ This could explain why interventions based on CTX increases have failed to prevent BMD loss post-denosumab.^42^ Tartrate-resistant acid phosphatase, an enzyme produced by osteoclasts, may better reflect early bone resorption, with levels rising before CTX and correlating more closely with BMD changes.^43^ Our findings of increased dipeptide levels, particularly phenylalanylleucine, beginning at 6 mo in the denosumab group and persisting to 24 mo, align with this observation. We and others have shown sustained RANKL elevations after denosumab initiation and withdrawal.^14^^,^^44^ Accumulation of osteoclast precursors during treatment and their reactivation via fusion into mature osteoclasts through “osteomorphs” has been proposed as a central mechanism of rebound bone resorption.^45^ Future research is needed to explore whether the identified dipeptides play a role in osteoclast differentiation or activity and whether fibrinogen-derived peptides contribute to bone metabolism regulation.

Our findings may have important clinical implications. Identifying novel serum peptides associated with bone metabolism, particularly COL1A1-OxP and DVP, underscores the potential to expand the current repertoire of biomarkers used to monitor skeletal health and therapeutic response. Both peptides demonstrated strong correlations with established ELISA-based markers of bone turnover, CTX and P1NP, while offering the additional advantage of being detectable through untargeted metabolomics. This approach enables multiplexed analysis and may allow for earlier and more nuanced detection of metabolic changes related to bone formation and resorption, potentially surpassing the capabilities of conventional antibody-based assays.

Notably, the consistent increase in dipeptides such as phenylalanylleucine in denosumab-treated patients and their close temporal alignment with known markers of rebound bone loss suggests these compounds could serve as early indicators of treatment discontinuation effects. Monitoring such biomarkers may enable timely clinical interventions, including initiating sequential therapies to mitigate bone loss following denosumab withdrawal. This is particularly relevant given the limitations of using CTX alone to guide treatment, as CTX elevations often lag behind actual bone degradation.^43^

Furthermore, the discovery of fibrinogen-derived peptides that show divergent profiles between treatment groups in the later phases posttreatment raises intriguing questions about the interplay between coagulation, inflammation, and bone remodeling. If validated, these peptides could provide mechanistic insights and emerge as novel therapeutic targets or systemic biomarkers relevant to broader skeletal health outcomes.

Our study has strengths. Data originate from a randomized clinical trial, ensuring balanced baseline characteristics, and the study population consisted of well-defined patients without osteoporosis or abnormal bone turnover. Baseline correction was applied throughout, allowing for the exclusion of features likely to represent false positives (eg, clusters 1 and 2). However, this approach may have excluded relevant findings, such as those in cluster 8. Including both corrected and uncorrected analyses provides a broader interpretive framework.

Nonetheless, several limitations should be acknowledged. As a post hoc analysis, the study was not originally powered for metabolomics, which may limit the ability to detect more subtle metabolic effects. Furthermore, we employed a deliberately conservative approach in the identification process, resulting in only 30 out of 83 significant features being confidently annotated. Although type I collagen is most abundant in bone, it is also present in soft tissues such as skin and muscle. While bone turnover typically occurs at a higher rate than in other tissues, we cannot rule out the possibility that the newly identified peptides, COL1A1-OxP and DVP, may originate from nonskeletal sources and may not be directly linked to bone formation or turnover. In addition, these potential biomarkers have not yet been validated using isotopically labeled standards or subjected to absolute quantification. Therefore, further validation in larger, independent cohorts and within a targeted biochemical framework is essential to clarify their specific role in bone metabolism during and after denosumab treatment.

In summary, metabolomics is an increasingly valuable tool in investigating osteoarthritis and osteoporosis.^16^^,^^46^ Our study demonstrates that serum metabolomics can uncover key biological processes underlying denosumab’s rebound effects and identify novel peptides, such as COL1A1-OxP and DVP, that may serve as biomarkers of bone metabolism. These markers, measurable via LC-HRMS, can complement or even replace conventional antibody-based assays, enabling more comprehensive and multiplexed monitoring of skeletal health. Beyond their diagnostic value, these findings suggest that metabolomics could play a pivotal role in identifying individuals at risk of adverse outcomes following denosumab discontinuation and inform the development of personalized follow-up strategies. However, further validation in larger, well-powered prospective cohorts is essential before these biomarkers can be translated into routine clinical applications.

## Supplementary Material

SupplementalFigure1_2_ziaf091

Supplemental_Table_1_As_Word_Text_ziaf091

SupplementalTable2_ziaf091

## Data Availability

According to the decision by the Swedish Ethical Review Authority and the Swedish Patient Data law, no other than aggregated data can be shared. This restriction also applies to anonymized/pseudonymized data since they are still considered sensitive. After a supplementary application by the responsible researcher, the Ethical Review Board may extend access to the dataset.
