# Supplementary figures and images for: Uncemented hip arthroplasty and denosumab: increased postoperative dipeptide concentrations and identification of potential new bone turnover biomarkers
Source: JBMR Plus. 2025 May 19;9(7):ziaf091. doi: 10.1093/jbmrpl/ziaf091 (PMC12202150; doi:10.1093/jbmrpl/ziaf091)

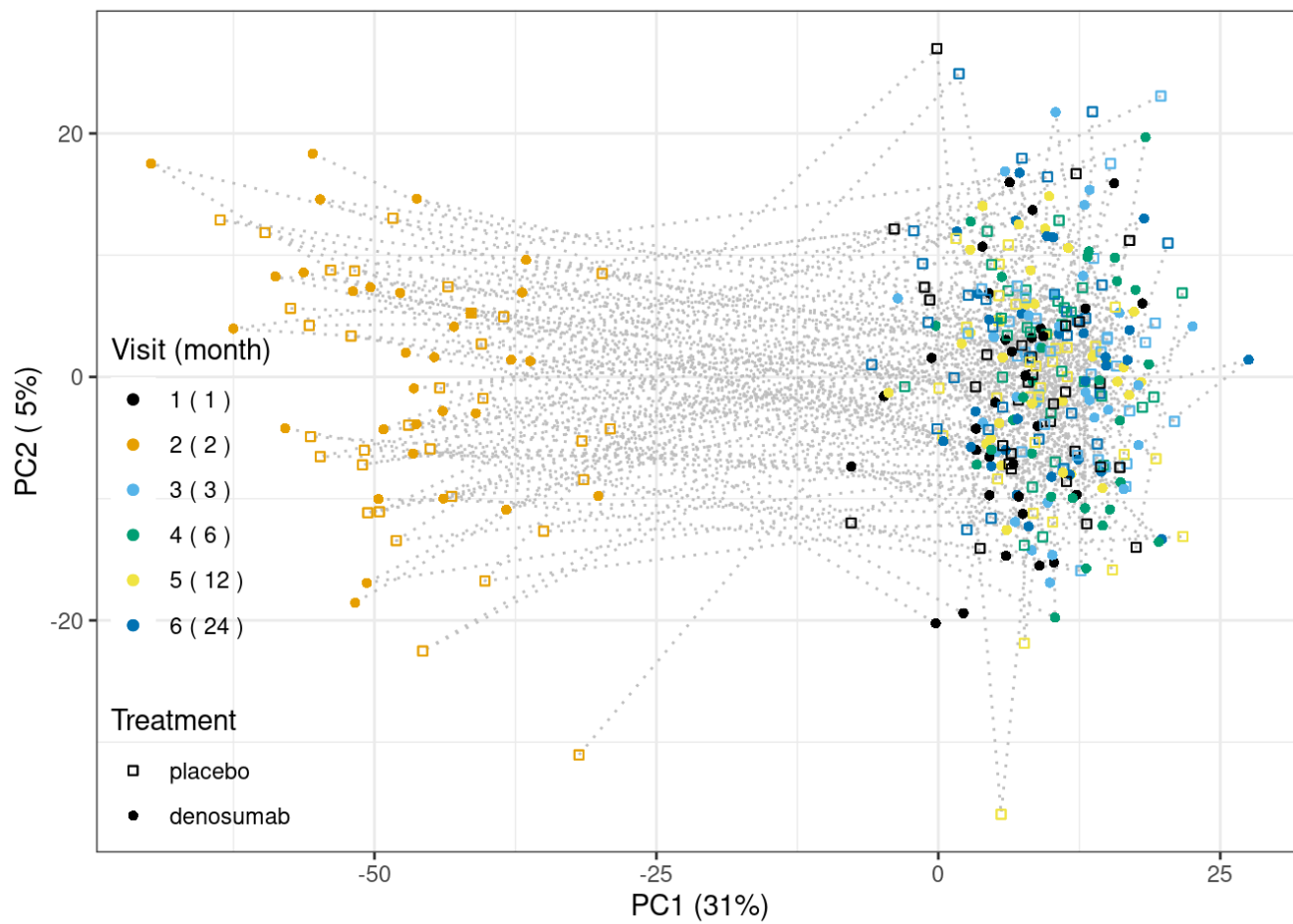

Supplement: SupplementalFigure1_2_ziaf091 [file supplementalfigure1_2_ziaf091.pdf]
