# Supplementary material for: Uncemented hip arthroplasty and denosumab: increased postoperative dipeptide concentrations and identification of potential new bone turnover biomarkers
Source: JBMR Plus. 2025 May 19;9(7):ziaf091. doi: 10.1093/jbmrpl/ziaf091 (PMC12202150; doi:10.1093/jbmrpl/ziaf091)
Supplement: Supplemental_Table_1_As_Word_Text_ziaf091 [file supplemental_table_1_as_word_text_ziaf091.docx]

**Supplemental Table 1**. Principal components (PC) and explained variance.

| **PC** | **Explained variance (%)** | **Global (p-value)** | **Treatment:Visit (p-value)** | **Treatment (p-value)** | **Visit (p-value)** | **Age (p-value)** | **Sex (p-value)** | **BMI (p-value)** |
| --- | --- | --- | --- | --- | --- | --- | --- | --- |
| PC4 | 4.52 | 4.48E-10 | 9.71E-1 | 9.99E-1 | 4.18E-14 | 9.80E-1 | 9.37E-1 | 9.99E-1 |
| PC13 | 1.54 | 5.71E-4 | 2.13E-1 | 9.92E-1 | 5.14E-5 | 9.69E-1 | 9.73E-1 | 9.69E-1 |
| PC18 | 1.11 | 9.01E-4 | 2.10E-1 | 9.95E-1 | 1.00E-4 | 9.53E-1 | 9.25E-1 | 9.95E-1 |
| PC7 | 3.23 | 1.76E-3 | 7.17E-1 | 9.89E-1 | 4.11E-5 | 9.81E-1 | 9.82E-1 | 9.72E-1 |
| PC2 | 6.88 | 3.5E-3 | 4.69E-2 | 9.90E-1 | 2.93E-3 | 9.58E-1 | 9.60E-1 | 9.68E-1 |
| PC6 | 3.60 | 4.41E-3 | 3.55E-1 | 9.81E-1 | 3.99E-4 | 9.14E-1 | 9.84E-1 | 9.73E-1 |
| PC1 | 11.08 | 7.57E-3 | 2.08E-1 | 9.83E-1 | 1.61E-3 | 9.73E-1 | 9.81E-1 | 9.78E-1 |
| PC12 | 1.72 | 1.05E-2 | 6.35E-1 | 9.88E-1 | 5.58E-4 | 8.98E-1 | 9.90E-1 | 9.82E-1 |
| PC19 | 1.03 | 1.41E-2 | 4.91E-2 | 9.89E-1 | 1.84E-2 | 8.81E-1 | 9.84E-1 | 9.58E-1 |
| PC15 | 1.38 | 4.12E-2 | 8.03E-2 | 9.95E-1 | 4.59E-2 | 9.01E-1 | 9.95E-1 | 9.89E-1 |
